# Ovarian Hormone Deprivation Reduces Oxytocin Expression in Paraventricular Nucleus Preautonomic Neurons and Correlates with Baroreflex Impairment in Rats

**DOI:** 10.3389/fphys.2016.00461

**Published:** 2016-10-13

**Authors:** Vitor U. De Melo, Rayssa R. M. Saldanha, Carla R. Dos Santos, Josiane De Campos Cruz, Vitor A. Lira, Valter J. Santana-Filho, Lisete C. Michelini

**Affiliations:** ^1^Department of Physiology, Federal University of SergipeSão Cristóvão, Brazil; ^2^Department of Health and Human Physiology, Obesity Research and Education Initiative, Fraternal Order of Eagles Diabetes Research Center, Abboud Cardiovascular Research Center, Pappajohn Biomedical Institute, University of IowaIowa, IA, USA; ^3^Department of Physiology and Biophysics, Institute of Biomedical Sciences, University of São PauloSão Paulo, Brazil; ^4^Department of Biotechnology, Federal University of ParaibaJoão Pessoa, Brazil

**Keywords:** baroreflex, oxytocin, arterial pressure, autonomic nervous system, ovariectomy

## Abstract

The prevalence of cardiovascular diseases including hypertension increases dramatically in women after menopause, however the mechanisms involved remain incompletely understood. Oxytocinergic (OTergic) neurons are largely present within the paraventricular nucleus of the hypothalamus (PVN). Several studies have shown that OTergic drive from PVN to brainstem increases baroreflex sensitivity and improves autonomic control of the circulation. Since preautonomic PVN neurons express different types of estrogen receptors, we hypothesize that ovarian hormone deprivation causes baroreflex impairment, autonomic imbalance and hypertension by negatively impacting OTergic drive and oxytocin levels in pre-autonomic neurons. Here, we assessed oxytocin gene and protein expression (qPCR and immunohistochemistry) within PVN subnuclei in sham-operated and ovariectomized Wistar rats. Conscious hemodynamic recordings were used to assess resting blood pressure and heart rate and the autonomic modulation of heart and vessels was estimated by power spectral analysis. We observed that the ovarian hormone deprivation in ovariectomized rats decreased baroreflex sensitivity, increased sympathetic and reduced vagal outflows to the heart and augmented the resting blood pressure. Of note, ovariectomized rats had reduced PVN oxytocin mRNA and protein expression in all pre-autonomic PVN subnuclei. Furthermore, reduced PVN oxytocin protein levels were positively correlated with decreased baroreflex sensitivity and negatively correlated with increased LF/HF ratio. These findings suggest that reduced oxytocin expression in OTergic neurons of the PVN contributes to the baroreflex dysfunction and autonomic dysregulation observed with ovarian hormone deprivation.

## Introduction

Cardiovascular diseases are the main cause of death in women (Mozaffarian et al., 2015). Menopause, characterized by a reduction in the circulating levels of the ovarian hormones progesterone and estrogen, is an important risk factor for cardiovascular diseases. More specifically, ovarian hormone deprivation has been shown to lead to hypertension, abnormal plasma lipids, endothelial dysfunction, elevated oxidative stress, autonomic imbalance and baroreflex impairment, which collectively result in high cardiovascular morbidity and mortality (Jensen et al., 1990; Taddei et al., 1996; Mercuro et al., 2000; Irigoyen et al., 2005; Flues et al., 2010).

It is well known that brainstem nuclei and the paraventricular nucleus of hypothalamus (PVN) are major sites for regulation of cardiovascular autonomic responses. A transient blood pressure rise activates neurons within the nucleus tractus solitarii (NTS), which stimulate parasympathetic neurons in the nucleus ambiguus (NA) and the dorsal motor nucleus of vagus (DMV) (Dampney, 1994). The NTS also projects and excites the GABAergic neurons in the caudal ventrolateral medulla (CVLM), which reduce the activity of sympathetic premotor neurons within the rostral ventrolateral medulla (RVLM) projecting to heart and vessels (Dampney, 1994) This information is continuously conveyed to pre-autonomic PVN neurons, via ascending cathecolaminergic afferents arising from NTS and CVLM, thus regulating their neurosecretory activity (Michelini and Stern, 2009; Sladek et al., 2015). Oxytocinergic (OTergic) pre-autonomic neurons within the dorsal cap, ventromedial and posterior PVN subnuclei are activated by the ascending afferents and project back to brainstem nuclei and spinal cord, thus modulating the autonomic circulatory control (Buijs, 1978; Michelini and Stern, 2009; Geerling et al., 2010; Cruz et al., 2013; Sladek et al., 2015). The physiological relevance of central oxytocin-dependent signaling in control of cardiovascular responses has been demonstrated by several studies. Augmented oxytocinergic drive to brainstem areas sensitizes the baroreceptor reflex control of heart rate facilitating bradycardic responses (Higa et al., 2002; Cavalleri et al., 2011).

Several types of estrogen receptors are expressed in the PVN. The nuclear estrogen receptor β (ER-β), a G protein-coupled estrogen receptor, is specifically expressed in OTergic neurons (Alves et al., 1998; Hrabovszky et al., 1998; Brailoiu et al., 2007); however, a functional link between ovarian hormones and central oxytocin signaling remains to be determined. Therefore, we hypothesized that ovarian hormone deprivation blunts oxytocin expression and signaling in pre-autonomic areas of the PVN, thus contributing to baroreflex impairment, autonomic imbalance and hypertension. In the present study, we used ovariectomized rats to investigate whether ovarian hormones are required for normal oxytocin mRNA expression and protein content in the posterior, ventromedial and dorsal cap subnuclei of the PVN. Conscious hemodynamic recordings were used to assess resting blood pressure, heart rate and autonomic modulation of heart and vessels.

## Methods

### Animals

All surgical procedures and experimental protocols (11/2014) were approved by the Ethics Committee on Animal Research of Federal University of Sergipe, in accordance with the guide for the care and use of laboratory animals published by National Institute of Health (National Research Council (US) Committee for the Update of the Guide for the Care and Use of Laboratory Animals, 2011). Thirty-three female Wistar rats (193 ± 5 g) were housed in propylene cages with controlled environmental temperature (22 ± 1°C), 12 h dark/light cycle and water and chow *ad libitum*. Animals were randomly divided into two groups and submitted to ovariectomy (OVX, *n* = 16) or sham surgery (SHAM, *n* = 17).

### Surgical procedures

At 10 weeks of age, rats were anesthetized (Ketamine: Fort Dodge IA, USA, 80 mg.kg^−1^ plus Xylazine: Fort Worth TX, USA, 12 mg.kg^−1^, *i.p*.) and an abdominal incision was made. Ovaries were then exposed and removed through the oviduct section. SHAM rats were submitted to the same procedures without ovaries' removal. Rats were treated with ketoprofen (Biofen 1%, 2 mg.kg^−1^; Biofarm, Jaboticabal, Brazil) and penicillin (Pentabiotico Veterinario 24,000 i.u.kg^−1^; Fontoura Wyeth, Sao Paulo, Brazil) and allowed to recover for 1 week (Irigoyen et al., 2005). Efficiency of ovariectomy was confirmed by analysis of vaginal smears collected for 4 consecutive days. Essentially, only rats that exhibited diestrus phase in all days were included in the OVX group. All animals allocated to the SHAM group were in a regular estrous cycle and were euthanized with the same age.

Eight weeks after OVX or SHAM surgeries, rats were anesthetized (ketamine, 80 mg.kg^−1^, Fort Dodge IA, USA, plus xylazine, 12 mg.kg^−1^, Fort Worth TX, USA, *i.p*.) and a polyethylene catheter was implanted (PE-10/PE-50, Intramedic, Becton Dickinson Company, Sparks, MD, USA) into the left femoral artery. Twenty-four hours after the procedure, rats are freely moving in their cages and did not exhibit signs of stress. The arterial catheter was then connected to a pressure transducer coupled to the preamplifier (FE221, Bridge Amp, ADInstruments, Bella Vista, NSW, Australia) and the recording system (Powerlab, ADInstruments, Bella Vista, NSW, Australia). Resting pulsatile and mean arterial pressure (AP) were continuously recorded for 30 min and processed using a dedicated software (LabChart 7, ADInstruments, Bella Vista, NSW, Australia). The inflection points of pressure signal were identified to generate beat-to-beat time series of mean arterial pressure (MAP), systolic arterial pressure (SAP), diastolic arterial pressure (DAP) and pulse interval (PI). Heart rate (HR) was calculated as 1/PI. To avoid any bias, all rats included in this study were submitted to the same procedures.

### Assessment of cardiovascular autonomic control

The analyses of PI and SAP variability were performed using the CardioSeries software (v2.4) as previously described (Oliveira et al., 2012). Beat-to-beat series were obtained from pulsatile arterial pressure and converted into discrete points every 100 ms, using cubic spline interpolation (10 Hz). Ten-minutes record of each rat was used for this analysis. Prior to the calculation of the spectral density, data was visually inspected and the non-stationary segments were disregarded. Data was then divided into half-overlapping sequential sets of 512 data points (51.2 s). Segments were windowed with a Hanning window and then the spectrum of each segment was calculated by the FFT algorithm. The PI spectrum, representing the variability of autonomic control of the heart, is composed by bands of very low frequency (VLF; <0.02 Hz), low frequency (LF; 0.2–0.75 Hz) and high frequency (HF; 0.75–3.0 Hz). These values are usually expressed in normalized (nu). LF and HF units, obtained through the division of respective LF and HF power by the total power minus VLF. VLF of PI represents humoral factors that influence heart rate, HF of PI indicates the cardiac parasympathetic modulation, while LF of PI is generally accepted as an index of cardiac sympathetic modulation and LF/HF ratio represents the sympatho-vagal balance to the heart (Malliani et al., 1991; Stauss, 2007; Oliveira et al., 2012). The SAP spectrum reflects arterial pressure variance and is quantified in mmHg^2^. Its VLF component is affected by myogenic vascular function, renin-angiotensin system and nitric oxide, LF of SAP represents the vasomotor sympathetic modulation plus endothelial nitric oxide modulation, while HF of SAP is mainly influenced by alterations in cardiac output coupled to changes in the venous return during respiration (Janssen et al., 1995; Heart rate variability: standards of measurement, physiological interpretation and clinical use. Task Force of the European Society of Cardiology and the North American Society of Pacing and Electrophysiology, 1996; Stauss, 2007).

Spontaneous baroreflex sensitivity (sBRS) was measured in the time domain using the sequence method (Rienzo, 1995). Beat-to-beat arterial pressure series were analyzed with the CardioSeries software (v2.4) to detect sequences of at least 4 beats with increased SAP followed by PI lengthening or decreased SAP with PI shortening that showed correlations greater than 0.85. The slope of the linear regression between SAP and PI was used as a measure of sBRS (mmHg/s).

### Tissue sampling

After the functional measurements, rats were deeply anesthetized with ketamine and xylazine (300 and 60 mg/kg, respectively, *ip*) leading to the respiratory arrest. Rats assigned to the PCR experiments were immediately subjected to transcardiac perfusion with saline solution (0.09%, 40 ml/min, 5 min) and decapitated to remove the brain, which was quickly transferred to dry-ice. A slice including the medial and caudal parts of the nucleus (from 1.40 to 2.30 mm caudal to bregma, 800–1000 μm) was taken at the hypothalamic level and immediately frozen for bilateral punching of the PVN. Samples were then stored at −80°C for subsequent analyses. Rats assigned to immunohistochemistry assay were subjected to transcardiac perfusion with Dulbecco's Modified Eagle's Medium (DMEM-Sigma, 40 ml/min, ~300 ml), followed by infusion of 4% paraformaldehyde in 0.01M PBS (pH 7.4, 40 ml/min, ~300 ml) and decapitated for brain removal. The brain was post-fixed in 4% paraformaldehyde for 48 h at room temperature, cryoprotected in Tris-PBS (10 mM Tris, 0.9% NaCl, 10 mM phosphate buffer, pH 7.4, containing 0.05% merthiolate) containing 20% sucrose at room temperature for 24–30 h, and then incubated in 0.01M PBS that contained 30% sucrose solution and stored at 4°C for 3–4 days before further processing.

### Quantitative real-time PCR

mRNA expression was assessed via quantitative real time PCR (qPCR). TRizol reagent (0.5 ml) was added to samples and RNA extraction was performed according to the manufacturer's instructions. After extraction, RNA was dissolved in 10 μl of DEPC water and stored at −80°C. Reverse transcriptase reaction was performed only after DNase I was added to samples, and first-strand cDNA synthesis was made with 1 μg RNA/reaction, using ImProm-II Reverse transcriptase (Promega, USA), according to the manufacturer's instructions. RNaseOUT was also present during this process and cDNA was stored at −20°C. qPCR was performed in the Applied Biosystems 7500 Fast Real-Time PCR System (ThermoFisher, California, USA) using Platinum SYBR QPCR Supermix-UDG and specific oligonucleotides for Oxytocin (OT, sense primer, TAGACCTGGATATGCGCAAG; antisense primer, CTCGGAGAAGGCAGACTCAG) and Glyceraldehyde 3-phosphate dehydrogenase (GAPDH, sense primer, GGGCAGCCCAGA ACATCAT; antisense primer, CCGTTCAGCTCTGGGATGAC). OT mRNA expression, normalized to GAPDH, was calculated using the ΔΔCt method (Pfaffl, 2001) and expressed as fold change in relation to values exhibited by the SHAM-operated rats. All reagents and oligonucleotides were purchased from Invitrogen (San Diego, CA).

### Immunohistochemical analyses

Sequential hypothalamic coronal sections (30 μm, −1.80 to −2.12 caudal to the Bregma) were obtained as previously described (Paxinos and Watson, 2006) using a cryostat (Leica CM 1850; Nussloch, Germany). Sections were collected in tissue culture wells with 0.01 M PBS and then incubated with 0.03% Triton X-100 and 10% normal donkey serum for 30 min. For the immunofluorescence assay, sections were incubated overnight with primary antibody (polyclonal guinea pig anti-oxytocin, 1:200,000 dilution; Bachem, Bubendorf, Switzerland), followed by a 2-h incubation with secondary antibody (donkey anti-guinea pig Cy3-labeled, 1:500 dilution; Jackson ImmunoResearch Laboratories, West Grove, PA) diluted in T-PBS containing 0.03% Triton X-100 at room temperature. Slices were placed on slides and mounted with a coverslip and SlowFade Gold anti-fade reagent. Specificity of the antibody was tested by processing side-by-side slices without the incubation with the primary or the secondary antibody.

### Image acquisition and analysis

OT immunoreactivity was captured using an epifluorescence microscope (Leica DMLB, Wetzlar, Germany; × 200 magnification) coupled to a digital camera (Axio-Cam HRC; Carl Zeiss, Vision GmbH, Aalen, Germany). Slides were visually inspected to localize the dorsal cap, ventromedial, posterior and magnocellular PVN subnuclei, and image analyses were performed using the ImageJ software (NIH) as previously described (Higa-Taniguchi et al., 2007; Cavalleri et al., 2011; Cruz et al., 2013). The relative OT density and integrated OT density were used as indexes of protein content in specific PVN subnuclei. Relative OT density was calculated as the ratio of the area occupied by the thresholded signal and the total area of interest, and expressed as a percentage (% of total area). Integrated OT density (in arbitrary units, AU) was obtained by the product of OT density and the signal intensity.

### Statistical analysis

Results were expressed as means ± SEM. Body mass was analyzed by two-way ANOVA followed by the Bonferroni *post hoc* test. Potential differences in OT mRNA and OT protein content within the PVN subnuclei, as well as hemodynamic parameters and baroreflex sensitivity were compared between SHAM and OVX rats and analyzed by unpaired *t*-test. Relationships between OT content within the ventromedial PVN and sBRS was assessed by the Pearson correlation coefficient. Differences were considered significant at *p* < 0.05.

## Results

### Body mass and autonomic cardiovascular control

Although there were no differences between groups in body mass at beginning of experiments, OVX rats gained significantly more weight than SHAM rats by the end of the study (~50% vs. ~26%, respectively; Table 1). OVX rats also exhibited higher basal MAP (*p* = 0.019) accompanied by a trend toward higher HR when compared to the SHAM group (*p* = 0.062, Table 1). PI variance, VLF, LF and HF in absolute units were similar in OVX and SHAM rats (*p* = 0.344, 0.667, 0.474 and 0.551, respectively); however, these rats exhibited reduced HF of PI (*p* = 0.015) and elevated LF of PI (*p* = 0.015) yielding an elevated LF/HF ratio (*p* = 0.015). SAP variance was higher in OVX vs. SHAM rats (*p* = 0.011), without significant differences between groups in the VLF, LF or HF of SAP (*p* = 0.059, 0.210 and 0.730, respectively). In addition, sBRS was largely reduced in OVX group when compared to SHAM (−59%, *p* = 0.005).

**Table 1:** **Body mass (before and after surgeries), baseline mean arterial pressure (MAP) and heart rate (HR) and cardiovascular autonomic evaluation in rats submitted to ovariectomy (OVX) or SHAM surgery**.

		**SHAM**	**OVX**
Body mass (g)	Before After	196 ± 2 248 ± 3 ^†^^†^^†^	191 ± 1 288 ± 7 ^†^^†^^†^^***^
**HEMODYNAMIC/AUTONOMIC EVALUATION**			
MAP (mmHg)		99 ± 3	109 ± 2^*^
HR (b/min)		353 ± 6	368 ± 3
PI variance (ms^2^)		27.84 ± 2.38	24.80 ± 1.81
VLF of PI (ms^2^)		12.40 ± 5.04	9.78 ± 2.48
LF of PI (ms^2^)		3.26 ± 1.57	5.79 ± 3.22
HF of PI (ms^2^)		11.50 ± 3.87	8.40 ± 3.04
LF of PI (nu)		22.50 ± 3.18	39.33 ± 5.14^*^
HF of PI (nu)		77.50 ± 3.18	60.66 ± 5.14^*^
LF/HF ratio		0.31 ± 0.05	0.77 ± 0.16^*^
sBRS (ms/mmHg)		1.76 ± 0.25	0.73 ± 0.11^**^
SAP variance (mmHg^2^)		13.56 ± 1.57	19.82 ± 1.23^*^
VLF of SAP (mmHg^2^)		4.17 ± 0.96	7.31 ± 1.15
LF of SAP (mmHg^2^)		5.13 ± 1.23	7.59 ± 1.39
HF of SAP (mmHg^2^)		4.38 ± 0.99	4.90 ± 1.05

Values are expressed as mean ± SEM. Body weight was evaluated in 16–17 rats/group and hemodynamic/autonomic measurements were made in 6–7 rats/group. VLF, LF, and HF represent the very low frequency, low frequency and high frequency bands of pulse interval (PI) and systolic arterial pressure (SAP) variance. sBRS, spontaneous baroreflex sensitivity. Significances (

* *P < 0.05)*,

** P < 0.01;

*** P < 0.001 are *vs. SHAM;

† *vs. before*.

### Effects of ovarian hormone deprivation on PVN OTergic neurons

**Figure 1:**
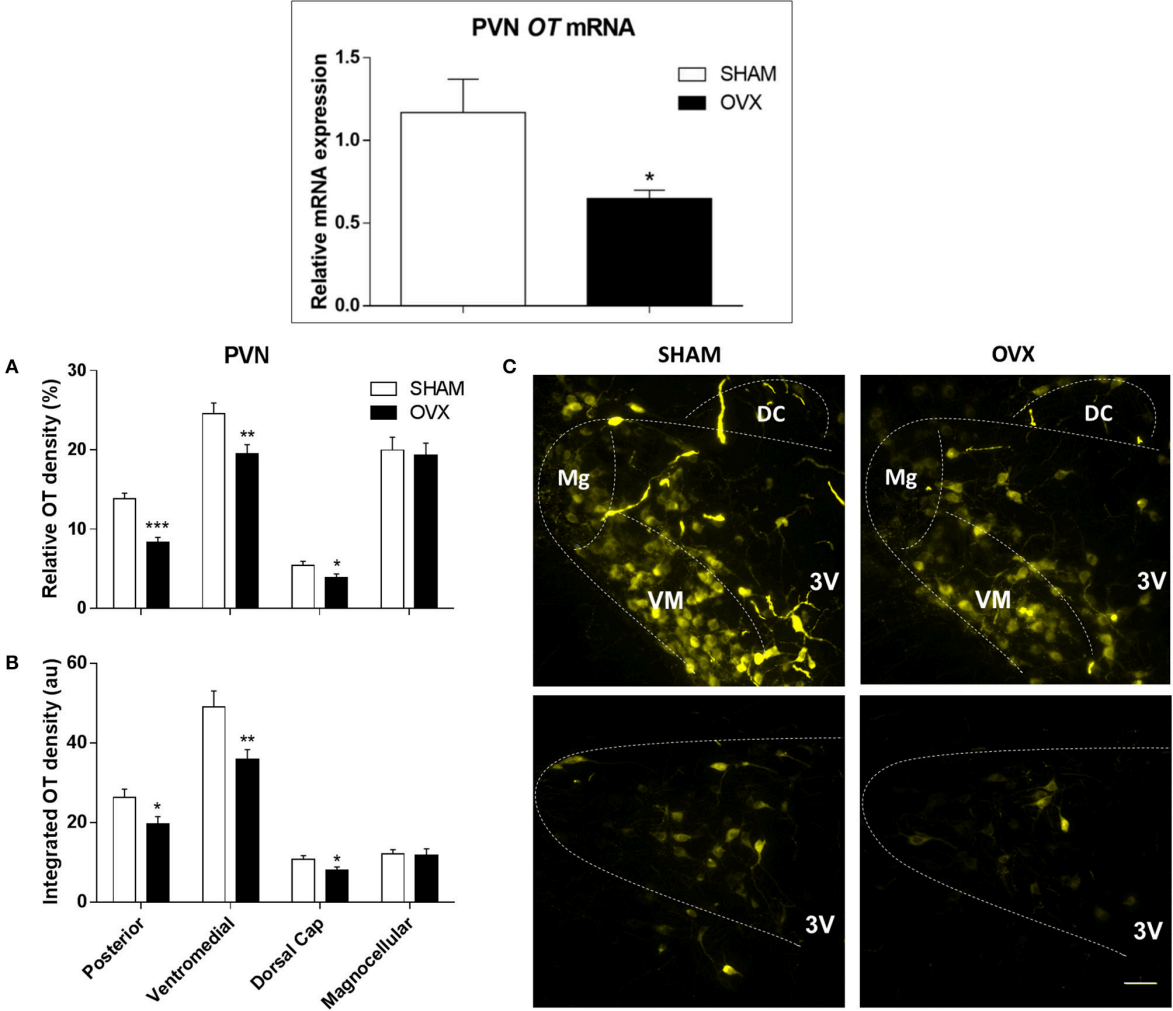
**Oxytocin (OT) gene and protein expression within the paraventricular nucleus of hypothalamus (PVN) in SHAM and OVX rats**. Upper panel: Comparison of relative OT mRNA content in SHAM and ovariectomized (OVX) rats (*n* = 6/group). Lower panels: Quantification of relative **(A)** and integrated OT density **(B)** within the posterior (post), ventromedial (VM), magnocellular (Mg) and dorsal cap (DC) PVN subnuclei of SHAM and OVX groups. Values are measured in 4–5 slices, 4 rats/group. Significances are (^*^P < 0.05; ^**^P < 0.01; ^***^P < 0.001 vs.) SHAM group. **(C)** Illustrates photomicrographs representative of OT immunoreactivity in the different subnuclei of the PVN in SHAM and OVX rats. 3rd ventricle (3V). Scale bar corresponds to 50 μm.

Relative OT mRNA expression in the PVN was 45% lower in OVX rats compared to SHAM rats (*p* = 0.030) (Figure 1, upper panel). Accordingly, ovarian hormone deprivation significantly reduced OT protein expression levels, quantified by both relative and integrated OT densities within the posterior (*p* = < 0.001 and *p* = 0.024, respectively), ventromedial (*p* = 0.009 for both comparisons) and dorsal cap (*p* = 0.041 and *p* = 0.029, respectively) PVN subnuclei (Figures 1A–C). In contrast, no differences were observed in relative and integrated OT densities in the magnocellular neurons of OVX rats compared to SHAM controls (*p* = 0.778 and *p* = 0.852, respectively) (Figures 1A–C).

**Figure 2:**
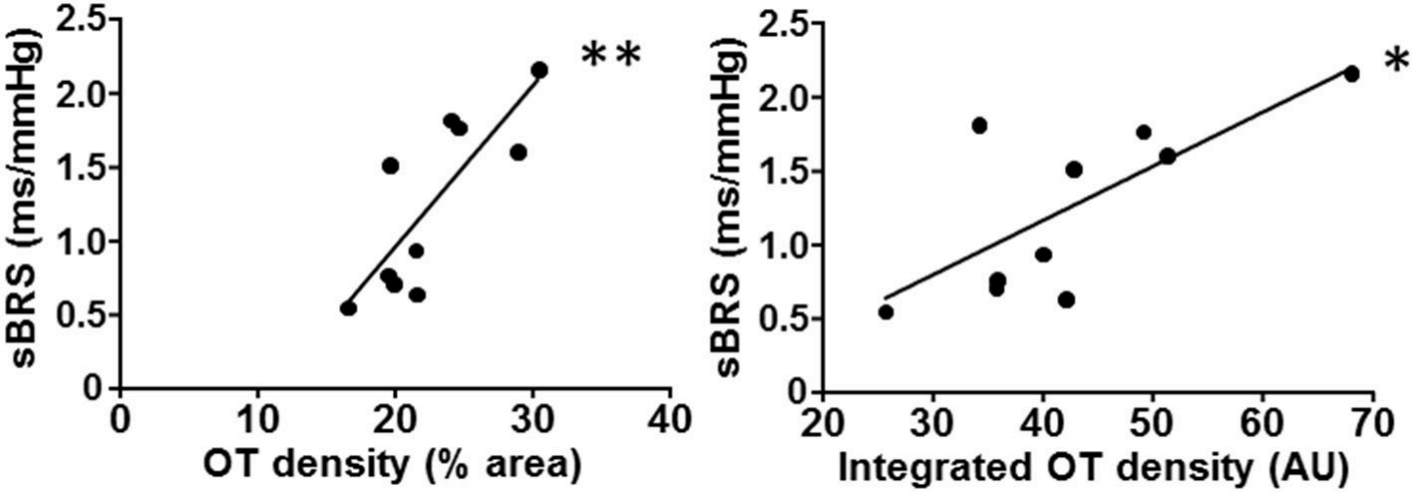
**PVN oxytocin (OT) immunoreactivity correlates with baroreflex sensitivity**. Decreased spontaneous baroreflex sensitivity (sBRS) is strongly correlated with the reduction of OT content within the ventromedial PVN (as measured by relative or integrated OT density) after ovarian hormone deprivation. Linear regression equations, correlation coefficients (r) and *P* values are shown in Table 2. ^*^P < 0.05 and ^**^P < 0.01 denote significant correlations.

Interestingly, reduced oxytocin protein levels within the posterior and ventromedial PVN subnuclei were strongly correlated with decreased autonomic control of the heart in OVX rats, as indicated by both decreased baroreflex sensitivity and increased LF/HF ratio (Figure 2, Table 2). Despite not reaching statistical significance, a similar relationship was also observed between OT expression levels at the dorsal cap and sBRS (Table 2).

**Table 2:** **Regression equations correlating OT immunoreactivity (OT***ir***, measured as relative density and integrated density in different PVN subnuclei) with spontaneous baroreflex sensitivity (BRS) and sympatho-vagal balance to the heart (LF/HF ratio) in rats submitted to SHAM and OVX surgery**.

	**Relative OT density (% área)**	**Integrated OT density (arbitrary units)**
**OT*ir* x BRS**		
*PVN ventromedial*	*Y* = 0.109 × −1.213^**^ r = 0.806 *P* = 0.005	*Y* = 0.037 × −0.319^*^ r = 0.730 *P* = 0.017
*PVN posterior*	*Y* = 0.193 × −0.886^***^ r = 0.945 *P* < 0.001	*Y* = 0.054 × +0.041^*^ r = 0.630 *P* = 0.049
*PVN dorsal cap*	*Y* = 0.247 × +0.110 *r* = 0.568 *P* = 0.087	*Y* = 0.091 × +0.409 *r* = 0.436 *P* = 0.207
*PVN magnocellular*	*Y* = 0.057 × +0.153 *r* = 0.181 *P* = 0.615	*Y* = −0.042 × +1.725 *r* = −0.184 *P* = 0.611
**OT*****ir*** × **LF/HF ratio**		
*PVN ventromedial*	*Y* = −0.037 × +1.372 *r* = −0.431 *P* = 0.213	Y = −0.009 × + 0.930 *r* = −0.288 *P* = 0.419
*PVN posterior*	Y = −0.095 × + 1.590^*^ r = −0.737 *P* = 0.015	Y = −0.008 × + 0.710 *r* = −0.142 *P* = 0.696
*PVN dorsal cap*	Y = −0.147 × + 0.082 *r* = −0.536 *P* = 0.110	Y = −0.044 × + 0.948 *r* = −0.336 *P* = 0.342
*PVN magnocellular*	Y = −0.018 × + 0.887 *r* = −0.091 *P* = 0.802	Y = 0.072 × −0.279 *r* = 0.501 *P* = 0.140

*OTir x BRS and OTir x LF/HF ratio correlations were made with 8–10 rats*.

* *(P < 0.05)*,

** (P < 0.01) and

*** *(P < 0.001) denote significant correlations*.

## Discussion

The present findings provide further support for a dysfunctional autonomic cardiovascular control caused by ovarian hormone deprivation. More specifically, our results demonstrate that lack of ovarian hormones impairs baroreceptor reflex control of heart rate, causes autonomic imbalance and increases blood pressure. Although several cellular and molecular mechanisms are likely involved, the present findings further indicate that reduced expression of oxytocin in OTergic neurons of the pre-autonomic PVN subnuclei are involved in the defective autonomic regulation observed. An important role for oxytocin in autonomic regulation has been previously documented (Mercuro et al., 2000; Cavalleri et al., 2011; Cruz et al., 2013). However, to our knowledge, this is the first study demonstrating that ovarian hormone deprivation reduces oxytocin gene and protein expression in pre-autonomic PVN neurons, and that these changes are closely correlated with autonomic dysfunction in ovariectomized rats.

The incidence of cardiovascular diseases is considerably lower in pre-menopausal women than in men. Nevertheless, this difference is mitigated by the reduction of circulating estrogen levels as women age (Becker and Corrao, 1990; Wenger et al., 1993; Tunstall-Pedoe et al., 1994; Golden et al., 2002). Consistent with our findings, previous studies have shown that estrogen promotes cardioprotection and metabolic homeostasis and that ovarian hormone deprivation, in addition to cardiovascular deficits, causes body weight gain with increased abdominal fat and reduced signaling in central nuclei that control appetite and satiety (Irigoyen et al., 2005; Flues et al., 2010; Lizcano and Guzmán, 2014). Oxytocin is an important peptide involved in the food intake control. Oxytocin or oxytocin agonist centrally administered decreased food consumption while oxytocin antagonist pretretment failed to increase chow intake (Olson et al., 1991; Mullis et al., 2013). In fact, weight gain induced by abnormal central oxytocin expression could *per se* contribute to baroreflex dysfunction and hypertension development (Skrapari et al., 2007; Re, 2009).

Previous studies indicate that ovarian hormone deprivation is able to reduce oxytocin mRNA expression in the brain, including the PVN (Miller et al., 1989; Patisaul et al., 2003). This study, however, is the first to indicate that lack of female hormones is specific to decrease oxytocin expression in preautonomic PVN subnuclei and that this effect is related to autonomic impairment.

Reduced baroreflex sensitity and increased sympathetic outflow to the heart are predictors of morbidity and mortality in several cardiovascular diseases (Billman et al., 1982; Becker and Corrao, 1990; Mercuro et al., 2000). Here, we observed significant reduction in sBRS coupled with increased sympathetic modulation to the heart and elevated blood pressure in rats deprived of ovarian hormones. These findings are in line with previous studies in ovariectomized rats, in which these changes were associated with augmented oxidative stress in the heart (Irigoyen et al., 2005; Flues et al., 2010). It should be noted that some studies did not find blood pressure changes and autonomic misbalance in ovariectomized rats (Nickenig et al., 1998; Dias et al., 2010). This inconsistency may be explained by the time that rats were exposed to ovarian hormone deprivation. In these studies, the ovariectomy lasted 3–5 weeks. To our knowledge, studies showing high blood pressure levels and dysautonomia were performed at least 8 weeks after ovariectomy induction (Hernández et al., 2000; Irigoyen et al., 2005; Flues et al., 2010 and the present set of data).

Our results together with previous studies suggest that deficient oxytocin expression within preautonomic PVN subnuclei may be an important mechanism of central autonomic deregulation, which contributes to cardiac oxidative stress and may lead to heart dysfunction. Future studies are necessary to test this proposition. In fact, heart disease is a major cause of morbidity and mortality in post-menopausal women (Mozaffarian et al., 2015).

Accumulating evidence indicate a critical role for preautonomic PVN OTergic neurons in the modulation of baroreceptor reflex control of heart rate. The nucleus tractus solitarii/dorsal motor nucleus of vagus (NTS/DMV) complex receives dense PVN OTergic projections, whose activation facilitates vagal outflow to the heart, thus improving reflex bradycardia during transient pressure increases (Buijs, 1978; Higa et al., 2002). It has also been shown that oxytocin released within the NTS/DMV during an acute bout of exercise reduces exercise tachycardia and causes resting bradycardia in trained rats (Braga et al., 2000; Higa-Taniguchi et al., 2009). Collectively, these observations indicate that adequate levels of oxytocin expression in PVN subnuclei are required for effective autonomic regulation of the cardiovascular system. In fact, our current results demonstrate reduced oxytocin expression within the ventromedial and posterior PVN and baroreflex impairment in OVX rats. It was shown that reduced oxytocin content, the neurotransmitter co-released with glutamate in those preautonomic neurons, blunts the activation of OTergic projections to dorsal brainstem areas (Piñol et al., 2014). Peters et al. (2008) also showed that activation of these projections augments glutamate release probability and the frequency of miniature excitatory post-synaptic currents in 2nd order NTS neurons while oxytocin antagonist pretreatment completely blocks this effect. Indeed, in a previous study in conscious rats we observed that oxytocin administration within the NTS/DMV area, mimicking the activation of the long-descending PVN oxytocinergic projections, augments the reflex bradycardia during baroreceptors loading, while its endogenous blockade reduces the bradycardic response (Higa et al., 2002). We also demonstrated that atropine, but not propranolol pretreatment, abrogates the augmentation of reflex bradycardia, indicating that improvement of baroreflex gain is mediated by oxytocin-induced increase in the vagal tonus to the heart (Higa et al., 2002; Michelini, 2007). The present set of data also showed strong positive correlations between OT content and baroreflex sensitivity. Although the correlations *per se* are not proof of causality, our results taken together with previous data on oxytocin and autonomic control strongly suggest that the reduction of PVN oxytocinergic drive in ovariectomized rats may be responsible for both the blunting of baroreflex sensitivity and the increased sympatho-vagal balance to the heart, as shown by the present set of data.

Heart rate variability has been widely accepted as an index of cardiovascular autonomic function. HF has always been associated to parasympathetic modulation; LF represents the sympathetic modulation, but there is evidence that parasympathetic component is also partially aggregated to this band (Appel et al., 1989; Burr, 2007). Some studies verified that LF of PI does not correlate with cardiac norepinephrine spillover and is very low in heart failure individuals (Adamopoulos et al., 1992; Guzzetti et al., 1995; Eisenhofer et al., 1996; Moak et al., 2007). However, the normalization procedures applied to absolute results yield values that are exchangeable across to different evaluation methods. Helpfully, presentation of data in normalized units mitigated several differences in the computed band power (Burr, 2007) and LF (in normalized units) is generally accepted as an index for sympathetic variability. In addition, our results corroborate previous studies that performed cardiac autonomic evaluation by pharmacological blockade (a gold standard method) in a similar protocol of ovarian hormone deprivation (Irigoyen et al., 2005; Flues et al., 2010).

OTergic neurons express β estrogen receptors (ER-β), which may control neuronal oxytocin gene/protein expression within PVN subnuclei (Alves et al., 1998; Hrabovszky et al., 1998). Stern and Zhang (2003) showed that preautonomic neurons within the posterior PVN subnucleus projecting to the rostral ventrolateral medulla exhibited high ER-β density and reduced excitability after ovarian hormone deprivation. It has also been shown that G-protein coupled estrogen receptors (GPERs) are located in several areas of the central nervous system, including the ventromedial and dorsal cap parvocellular neurons (Brailoiu et al., 2007). GPERs have been shown to co-localize with oxytocin in magnocellular PVN and supraoptic neurons (Brailoiu et al., 2007). Whether a direct physical interaction between GPERs and oxytocin in PVN subnuclei indeed occurs, and how such interaction may potentially contribute to the maintenance of oxytocin levels (e.g., via stabilization) remain to be determined. A potential transcriptional regulation of the oxytocin gene by ER-β may also be in place and deserves further investigation.

In summary, our results showed that ovarian hormone deprivation decreases oxytocin gene and protein expression within PVN pre-autonomic neurons involved in circulatory control. The observed deficits in OTergic modulation were accompanied by reduced vagal and increased sympathetic modulation to the heart and augmented SAP variability. Finally, oxytocin content in the PVN was closely correlated with autonomic control of the heart suggesting that depressed hypothalamic OTergic modulation significantly contributes to the cardiovascular deficits observed in ovarian hormone deprivation.

## Author contributions

VUD, RS, and CD: Performed the experiments; VUD and RS: Analyzed the data; VUD, VL, JD, and LM: Wrote, edited and revised the manuscript; VL, VJD, and LM: Approved the final version of the manuscript.

## Funding

This work was supported by Coordenação de Aperfeiçoamento de Pessoal de Nível Superior (CAPES/ Proc BEX 6292/15-1), Fundação de Apoio à Pesquisa e Inovação Tecnológica do Estado de Sergipe (FAPITEC-SE) and Fundação de Amparo à Pesquisa do Estado de São Paulo (FAPESP, proc 2011/51410-9). LM is a Research Fellow of CNPq.

### Conflict of interest statement

The authors declare that the research was conducted in the absence of any commercial or financial relationships that could be construed as a potential conflict of interest.
